# Case report: From monkeypox pharyngitis to myopericarditis and atypical skin lesions

**DOI:** 10.3389/fcvm.2022.1046498

**Published:** 2023-01-05

**Authors:** María Ascensión Sanromán Guerrero, Elena Hernández Sánchez, Belén de Nicolás Ruanes, Pablo Fernández-González, Sonia Antoñana Ugalde, Alejandra González Leal, Marcelo Sanmartín Fernández, Jose Javier Alarcón Rodríguez, Laura Martinez Garcia, Rosa Escudero, Maria Ángeles Fernández Méndez, Jose Luis Zamorano Gómez, Beatriz Montero Llorente, Maria Jesús Vivancos-Gallego

**Affiliations:** ^1^Department of Cardiology Medicine, Hospital Ramón y Cajal, Madrid, Spain; ^2^Department of Dermatology, Hospital Ramón y Cajal, Madrid, Spain; ^3^Department of Radiology, University Hospital Ramón y Cajal, Madrid, Spain; ^4^Department of Microbiology, University Hospital Ramón y Cajal and IRYCIS, Universidad de Alcalá, CIBERESP, Madrid, Spain; ^5^Department of Infectious Diseases, University Hospital Ramón y Cajal and IRYCIS, Universidad de Alcalá, CIBERINFEC, Madrid, Spain; ^6^Department of Clinical Pharmacology, Hospital Ramón y Cajal, Madrid, Spain

**Keywords:** myocarditis, pericarditis, tecovirimat, monkeypox, Lake Louise criteria, plasma

## Abstract

**Background:**

A global outbreak of the human monkeypox virus (HMPXV), first identified in May 2022, was declared a health emergency of international concern on 23 July 2022. Before the global outbreak, monkeypox cases were mostly confined to central and west African countries, where this virus is prevalent. Close contact, mainly sexual contact, is supposed to be the main route of transmission, and it is remarkable that the incidence is higher in men who have sexual relationships with other men.

**Case summary:**

A 40-year-old Caucasian man arrived at the emergency department complaining of oppressive epigastric pain extending to the chest after a diagnosis of pharyngitis, which was suspected to be caused by the human monkeypox virus. Based on the clinical symptoms, physical examination, serum cardiac biomarkers, and electrocardiographic findings, he was diagnosed with myopericarditis. The real-time PCR for human monkeypox in skin lesions, urine, plasma, and the oropharyngeal swab was positive. The peak of troponin I was 20.6 ng/ml, and the electrocardiogram showed an upward concavity in the ST segment in diffuse leads, which was in agreement with the previous diagnosis. The presence of edema, subepicardial, and myocardial late gadolinium enhancement, and increased values on T1 mapping in the cardiac MRI were in agreement with the diagnosis of myopericarditis. Antiviral treatment with tecovirimat was started with excellent tolerability. After 6 days, the patient recovered and was discharged.

**Discussion:**

To our knowledge, this is one of the first reported cases of myopericarditis due to human monkeypox infection, which was confirmed by a cardiac MRI following modified Lake Louise criteria. The short span between the onset of the mucocutaneous symptoms and the myocardial damage suggests a pathogenic association. Furthermore, the active viral replication in plasma samples and the negative results on real-time PCR for other viruses support this clinical association.

## Introduction

Before April 2022, human monkeypox virus (HMPXV) infection had mostly been reported in Africa, where it was an endemic disease. The World Health Organization declared the HMPXV outbreak a public health emergency of international concern (PHEIC) on 23 July 2022. Currently, numerous cases occur worldwide after close or sexual contact, which is supposed to be the main transmission route. Skin lesions are detected in 95% of the affected people, influencing the anogenital area the most, followed by the arms and trunk in 50% of the cases. Mucosal lesions are reported in 41% of the cases. To date, gay or bisexual men are the most affected population group, suggesting the amplification of the spreading of the disease in sexual networks (1, 2).

Of the 528 cases worldwide, complications associated with HMPXV infection have been reported: one case of epiglottitis and two cases of myocarditis. Only 5% received specific treatment for HMPXV: tecovirimat (2%), cidofovir (2%), and vaccinia immune globulin (<1%) (1).

In this study, we report a case of myopericarditis after pharyngitis caused by an HMPXV infection, followed by the onset of atypical skin lesions. Without the requirement for vasoactive pharmaceutical treatment, a benign clinical course was observed. Nevertheless, tecovirimat was administered during hospitalization for heart damage, demonstrating its safety and a complete resolution of symptoms. In conclusion, despite the need for further research, this antiviral treatment is increasingly recommended for use in this clinical setting of heart damage.

## Case report

We present the case of a 40-year-old Caucasian man who sought emergency medical attention on 26 August 2022 due to odynophagia, a swollen right submandibular lymph node, cervical pain, and a fever of up to 38°C. He reported having had high-risk sexual contact (exclusively oral intercourse) 2 weeks before the onset of the symptoms. His male partner was diagnosed with genital HMPXV 5 days later with a positive real-time PCR for *orthopoxvirus*. Until that moment, our patient was asymptomatic. He had previously been diagnosed with human papillomavirus condyloma, for which he had been treated with cryotherapy and imiquimod. There were no known drug allergies. The patient lived in a suburban area of Madrid with his mother and no animals. He had never been vaccinated for smallpox. Informed consent was given prior to writing and publishing this case report.

**Figure 1:**
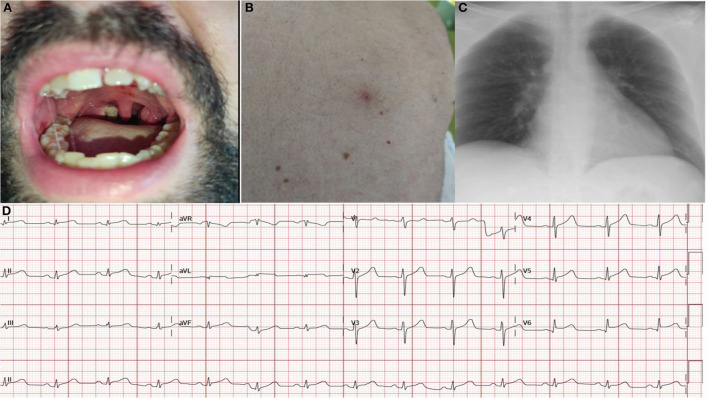
Skin lesions **(A, B)**, chest x-ray **(C)** at admission and electrocardiogram at admission **(D)**. At admission, the electrocardiogram revealed sinus rhythm with diffuse ST elevation with upward concavity in leads (I, II, III, AVF, V4, V5, and V6) and diffuse depressed PR segment.

The physical exam revealed swollen tonsils and uvula. Multiple coalescing ulcerations covered the right tonsil with a necrotic base and confluent lesions, which showed a necrotic center and white margins (Figure 1A). No anogenital or skin lesions were present at this initial visit. During his stay at the hospital, a small number of millimetric umbilicated pustules with an erythematous base on the trunk and the proximal region of the limbs also appeared (Figure 1B).

With verbal consent, swabs from nasopharyngeal, rectal, and urine samples were taken by the Dermatology Department at the Emergency Room (ER). Given that the patient did not present alarming signs, he was prescribed amoxicillin and anti-inflammatory drugs (ibuprofen) and was discharged.

Two hours later, he suddenly woke up at home, in his bed, due to an oppressive epigastric pain extending to the chest. The pain lasted approximately one and a half hours and prompted his return to the ER. At the time of admission, the temperature was 36.3°; blood pressure, 102/70 mm Hg; heart rate, 75 beats per minute; and basal oxygen saturation, 95%. There were no heart murmurs in the cardiac auscultation. No signs of edema were observed.

The chest x-ray revealed no significant findings (Figure 1C). The electrocardiogram (ECG) on arrival showed a diffuse ST elevation with an upward concavity in leads (I, II, AVL, V4, V5, and V6), a negative T wave in III, and a diffusely depressed PR segment (Figure 1D). The initial troponin I determination was 9.7 ng/ml (Architect Abbot), and the C-reactive protein level was 10.5 mg/L (normal value < 5 mg/L). The transthoracic echocardiogram revealed a preserved left ventricular ejection fraction. No regional wall abnormalities, no significant valve diseases, and no pericardial effusion was observed (Supplementary Video 1).

The patient was later admitted to the coronary care unit; he remained asymptomatic and hemodynamically stable. Chest pain was controlled with intravenous anti-inflammatory drugs (50 mg of dexketoprofen every 8 h) and 0.5 mg of colchicine every 12 h. No more drugs were administered. Forty-eight hours later, the HMPXV infection was confirmed by the Microbiology Department at our hospital. Samples sent for testing included urine and plasma; swabs were taken from a suspected skin lesion, and rectal and pharyngeal samples were collected. A microbiological diagnosis was made by real-time PCR (3). In addition, he was screened for sexually transmitted infections (STIs), including *Chlamydia trachomatis* and *Neisseria gonorrhoeae*, in a pharyngeal swab, a rectal swab, and a first-void urine sample, as well as HBV, HIV, HCV, and syphilis serology, which came back negative.

During admission to the coronary care unit, the Infectious Diseases team monitored the patient, and other potential causes of acute myopericarditis were discarded. Differential diagnoses included viral and bacterial infections, non-infectious causes such as drug abuse, hypersensitivity reactions, and systemic disorders.

**Figure 2:**
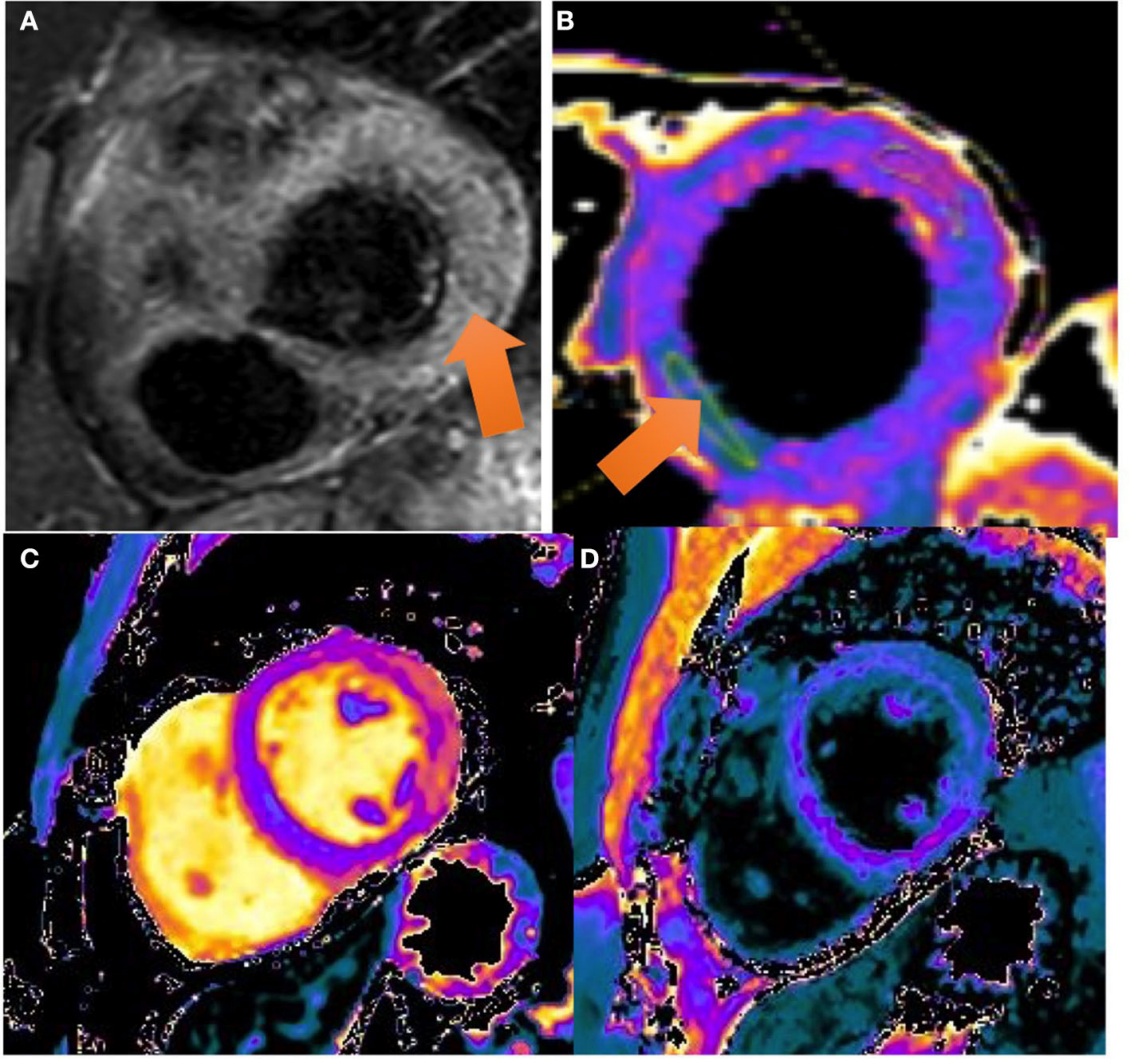
CMR images: the balanced FFE (fast field echo) sequences acquired along the short axis **(A, B)** and two chamber **(C)** and four chamber **(D)** views demonstrate myocardial late gadolinium enhancement with epicardial predominance in the lateral and inferior wall and mesocardial predominance in the septum and anterior wall (view arrows).

**Figure 3:**
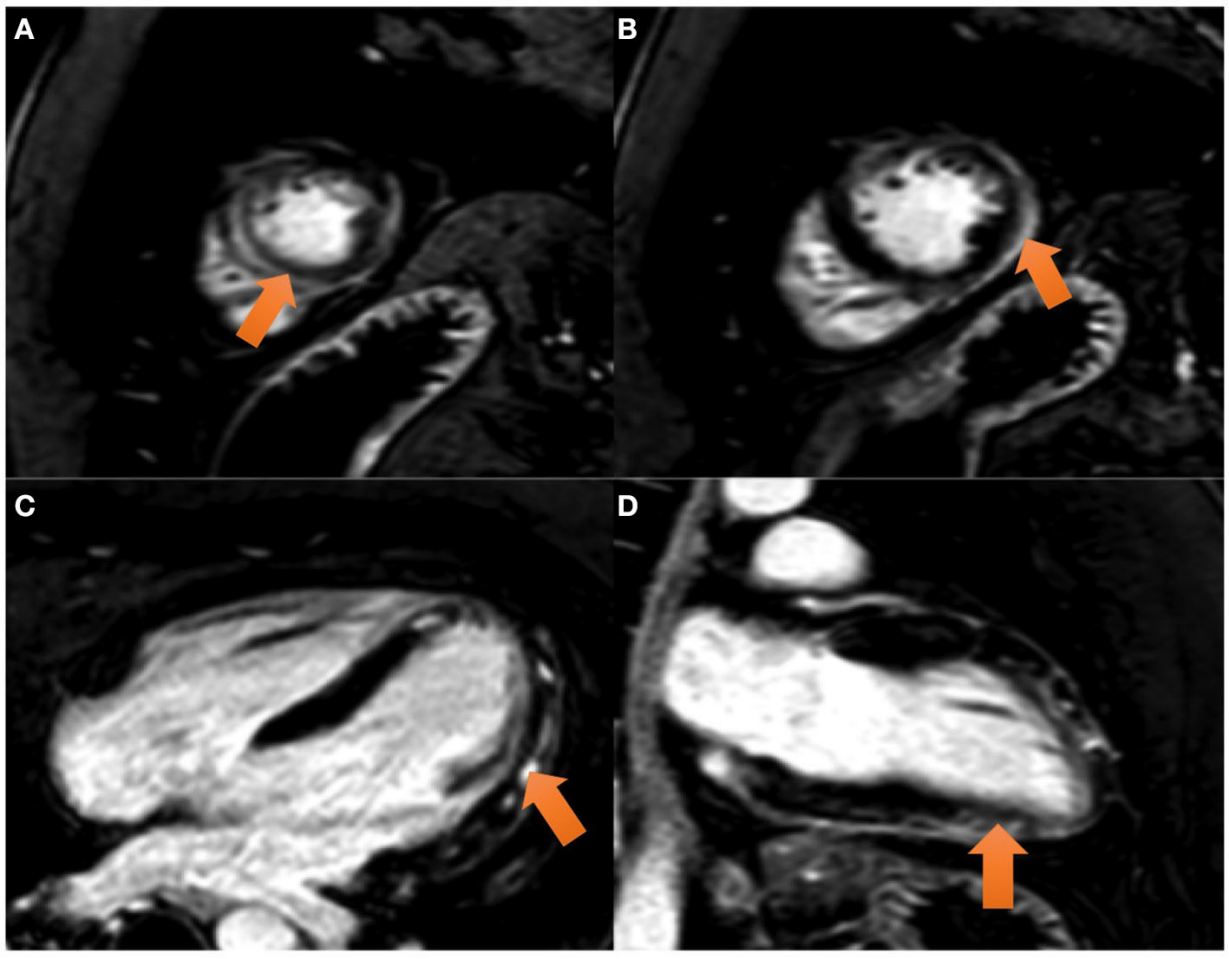
CMR images: **(A)** T2 STIR sequence: a high ratio (> 2) is seen in the intensity between the myocardium and the skeletal muscle compatible with a generalized edema. Color-coded image of T2 mapping in LV mid-chamber. The arrow marks an increased T2 value (T2 = 60 ms) in the lateral and inferolateral wall. **(B)** Color-coded image of T2 mapping in LV mid-chamber. The arrow marks an increased T2 value (T2 = 60 ms) in the lateral and inferolateral wall. **(C, D)** T1 mapping and T1 enhanced mapping, respectively. In T1 mapping, extensive area of increased T1 value (view pink and orange areas) in lateral and inferolateral walls. On quantitative analysis, values on the native T1 map were 1,160 ms (normal value 1,025–1,075 ms). In T1 enhanced mapping, these areas area correlated with increased extracellular volume (ECV).

Cardiac Magnetic Resonance (CMR) performed on day 6 showed a preserved left ejection fraction (56%) with no regional wall abnormalities. An increased myocardial and pericardial signal intensity on the T2 STIR sequence was suggestive of edema (Figure 2A). On the T1 mapping, an average native T1 of 1,160 ms was observed, and on the T2 mapping, an average T2 of 60 ms was observed. In addition, the quantitative assessment of the cardiac ECV (extracellular volume) was 35%. The T1 and T2 mapping values were measured on a 1.5 Tesla MR Philips machine within a single breath-hold using a modified Look-Locker with inversion recovery (MOLLI) and gradient and spin echo (Gra-SE) sequences, respectively. Normal native myocardial T1 values lie between 1,025 and 1,075 ms, whereas T2 values lie between 50 and 57 ms (internally validated). A color-coded image of T2 mapping (Figure 2B), native T1 (Figure 2C), and enhanced T1 mapping (Figure 2D) were also performed. Late gadolinium enhancement (LGE) images, obtained by using a balanced fast field echo (FFE) sequence, showed subepicardial and mesocardial enhancement of the myocardium and mild signs of pericarditis, which were considered consistent with acute myopericarditis (Figures 3A–D). The patient met all modified Lake Louise criteria for myocarditis: T2 criteria (myocardial edema shown on a T2 STIR sequence and T2 mapping) and T1 criteria (non-ischemic myocardial injury shown on an LGE sequence and T1 mapping). Signs of mild pericarditis were also exhibited.

According to the CMR results, the myocardial injury and inflammatory markers, as well as the remarkable electrocardiographic findings, supported the diagnosis of acute myopericarditis. Moreover, the temporary association with an active HMPXV infection and ruling out other possibilities of causes of myopericarditis suggested a myopericarditis associated with an *orthopoxvirus* infection. Therefore, a myocardial biopsy or coronariography was not required.

**Figure 4:**
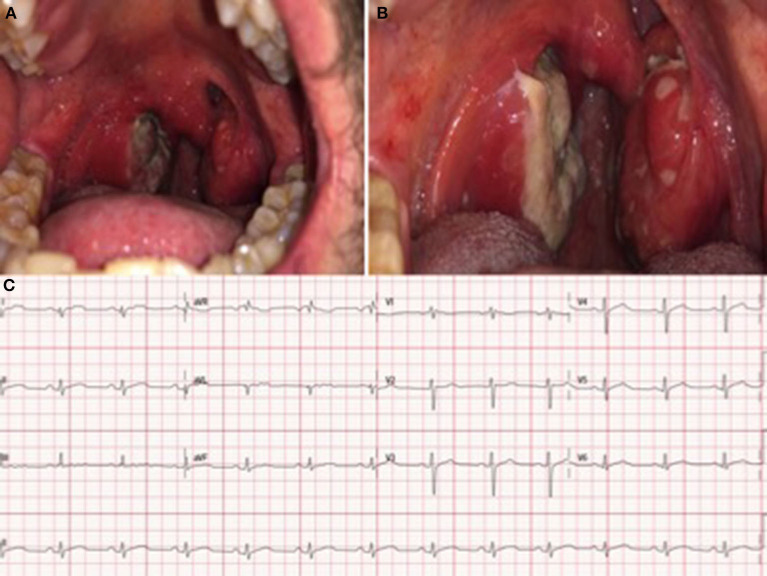
**(A)** Enlargement and coalescence of oral ulcerations on the tonsils and uvula 48 and 96 h after the diagnosis, respectively **(B)**. **(C)** Electrocardiogram at discharge: Sinus rhythm without significant findings.

As potential HMPXV infection complications (systemic involvement and myocardial damage) occurred in this patient, requiring hospitalization, tecovirimat was requested from a drug agency. Following the Infectious Diseases Department's instructions, the treatment started 48 h after admission: 600 mg of oral Tecovirimat every 12 h for 14 days. No adverse events were reported. During his stay at the hospital, the previous electrocardiographic findings became normal (Figure 4A), whereas the oropharyngeal lesions increased in size and coalesced into large ulcerations located on both tonsils and the uvula (Figures 4B, C).

After the resolution of the symptoms and the improvement in the inflammatory response and the myocardial injury markers (Table 1), the patient was discharged with close monitoring and follow up by both the Cardiology, Infectious Diseases, and Dermatology departments. The patient's last visit was on 5 October and he had completely recovered. Transthoracic echocardiogram and pharyngeal examination were normal without swollen submandibular adenopathy, and he remained asymptomatic. Pharyngeal samples for HMPXV tested positive on 5 September and 12 September and finally negative on 20 September. A new CMR will be performed 6 months after the episode, approximately on 20 March 2023.

**Table 1:** Trajectory of biomarkers.

	**Day 1**	**Day2**	**Day 3**	**Day 4**	**Day 6**
CRP (mg/l)	84.4	93.9	110.5	74.7	37.6
Tn-I (ng/ml)	20.6	11.8	4.8	0.7	0.1
WBC (10^∧^3/ml)	9.36	9.92	11.10	11.80	11.40
Fibrinogen (mg/dl)	502,10	300	N/A	N/A	N/A
BNP (pg/ml)	< 16	N/A	N/A	N/A	N/A

## Discussion

We present a case of myopericarditis associated with HMPXV infection treated with tecovirimat. Notably, four cases have been previously published, and only one of them was also treated with antiviral therapy (1, 2, 4–6).

This case reveals four aspects worth highlighting: an atypical clinical presentation, the frequent positive samples for HMPXV from different locations, a well-documented myopericarditis as an unusual complication, and the use of tecovirimat with a good clinical course.

The clinical signs of the HMPXV are frequently reported as cutaneous or genital lesions (1, 2). We observed some differences: pharyngeal involvement with large lymphadenopathy was a prominent initial finding, followed by myopericarditis prior to the rare atypical rash with minute pustular lesions.

The detection of HMPXV DNA in a pharyngeal swab sheds light on the transmission mechanisms of the infection, and the positive HMPXV DNA in plasma samples deserves special consideration since it can explain the systemic findings and myocardial involvement.

In this case, the diagnosis of myopericarditis was confirmed by cardiac magnetic resonance following modified Lake Louise criteria. Even though the temporal association does not prove causality, the short span between the onset of the mucocutaneous symptoms and the myocardial damage suggests a pathogenic association. Furthermore, the negative results with real-time PCR regarding other viruses commonly related to myocarditis and oral lesions support this clinical association. As previously mentioned, active viral replication in plasma samples was shown. This strengthens the systemic viral presence and possible heart damage (7, 8). This report highlights myopericarditis as a potential complication of monkeypox and aims to raise clinicians' awareness.

The clinical course of the HMPXV infection has been uniformly benign in most patients (1, 2). An antiviral treatment, tecovirimat, prevents the dissemination of the HMPXV in the host by inhibiting the activity of the orthopoxvirus VP37 envelope-wrapping protein, thereby preventing the formation of competent enveloped virions and virus particles from exiting human cells (9).

Tecovirimat has been recently approved (January 2022) for treating systemic symptoms of the monkeypox infection and is the sole treatment approved by the EMA to date. It may be indicated in severe forms of the disease (myocarditis, encephalitis, and other conditions needing hospital admission), immunocompromised patients, or patients presenting with mucocutaneous lesions with an atypical location and poor pain control (10–12).

Moreover, although we do not have conclusive evidence on the use of tecovirimat for monkeypox-related myopericarditis, it appears to be safe, as it led to clinical recovery (13, 14).

## Limitations

There is a lack of evidence regarding the pathophysiology, mechanisms, and efficacy of available antiviral treatments in HMPXV myopericarditis. These data are still emerging.

According to the available clinical evidence of myopericarditis due to other viral agents, the two most plausible mechanisms by which HMPXV can lead to myocarditis are direct damage to cardiomyocytes due to active replication of *orthopoxvirus* or muscle damage secondary to the autoimmune mechanism by molecular mimicry with viral proteins.

Similarly, previous vaccine studies against smallpox described myopericarditis cases as rare complications after vaccination (15–17). Mechanisms proposed to explain vaccine-related myopericarditis were damage that are directly attributable to the virus as the smallpox vaccine is a live-attenuated virus or an immune-mediated injury vaccine due to immune activation with peaks on days 8–9 (18–20).

The diagnosis of myopericarditis is challenging, but based on the clinical features of fever, chest pain, diffuse upward concave ST elevation, elevated inflammatory markers, the findings in the CMR, and positive *orthopoxvirus* DNA on a plasma sample, the first mechanism is more plausible.

Pathological confirmation of myopericarditis due to monkeypox with cardiac biopsy probably would have contributed to clarifying the diagnosis, but due to its inherent risks and lack of benefit in this patient, it was not pursued.

## Conclusions

Here, we present a case of mild myopericarditis with a favorable clinical course, which is consistent with previous reports. Based on our experience, tecovirimat may be beneficial in this setting; however, further research is needed in this field.

## Patients perspective

Since his clinical admission, the patient has been involved in all clinical decisions concerning him and is in agreement with all therapeutic methods and different alternatives. He has always been grateful for the treatment he received and was happy to be at the disposal of every scientific contribution.

On 9 September 2022, a reexamination was carried out on the patient's progress and clinical response to tecovirimat. A complete remission of the oral ulcers was observed. The submandibular right lymph node had also been progressively diminishing in size since the third day of starting the treatment with tecovirimat. The health status was excellent; thus, the treatment with tecovirimat was continued for 14 days. We observed no adverse or unanticipated events.

## Data availability statement

The raw data supporting the conclusions of this article will be made available by the authors, without undue reservation.

## Ethics statement

The patients/participants provided written informed consent to participate in this study.

## Author contributions

MAS, ES, AL, SU, MV-G, BR, and MSF wrote this clinical report. JR and MM provided and interpreted CMR images. MSF, SU, AL, MAS, ES, RE, MV-G, BR, PF-G, and JZ offered clinical assistance to the patient. LM performed the real time PCR in plasma. All the authors reviewed the manuscript. All authors contributed to the article and approved the submitted version.

## Appendix

**Table:** Timeline.

**12 August 2022**	**High-risk sexual contact with a male partner**.
16 August 2022	Exploration by a dermatologist due to high-risk sexual contact with a partner. Diagnosed of MPX by reactive polymerase chain reaction assay. Asymptomatic patient. No swabs or samples were taken.
26 August 2022	Odynophagia, fever, right submandibular adenopathy, cervical pain. Swollen tonsils and ulcerative lesions on the right tonsil. Anti-inflammatory drugs and amoxicillin were prescribed.
27 August 2022	Chest pain elevated cardiac markers. Diagnosis of myopericarditis. Anti-inflammatory treatment was initiated, and colchicine was.
28 August 2022	Tecovirimat started.
1 September 2022	Cardiac magnetic resonance was performed. Hospital discharge.
7 September 2022	Blood test. No blood test abnormalities.
9 September 2022	Follow-up by the Infectious Diseases Department. No side effects with tecovirimat. Almost completely recovered. Persistently swollen submandibular adenopathy.
10 September 2022	End of tecovirimat.
18 September 2022	End of quarantine.
20 September 2022	Pharyngeal samples for HPMX were taken on and were negative.
5 October 2022	The patient was fully recovered and asymptomatic.
